# Effects of Synbiotic Supplementation on Metabolic Syndrome Traits and Gut Microbial Profile among Overweight and Obese Hong Kong Chinese Individuals: A Randomized Trial

**DOI:** 10.3390/nu15194248

**Published:** 2023-10-02

**Authors:** Susana Lauw, Nelson Kei, Po Lam Chan, Tsz Kwan Yau, Ka Lee Ma, Carol Ying Ying Szeto, Janice Su-Chuen Lin, Sunny Hei Wong, Peter Chi Keung Cheung, Hoi Shan Kwan

**Affiliations:** 1 Food and Nutritional Sciences Program, School of Life Sciences, Faculty of Science, The Chinese University of Hong Kong, Hong Kong SAR, China; susana.lauw@link.cuhk.edu.hk (S.L.); nelsonkei@link.cuhk.edu.hk (N.K.); kellyma@link.cuhk.edu.hk (K.L.M.); petercheung@cuhk.edu.hk (P.C.K.C.); 2 Food Research Centre, The Chinese University of Hong Kong, Hong Kong SAR, China; polamchan@cuhk.edu.hk; 3 HSK GeneTech Limited, Hong Kong SAR, China; carol@hskgene.com; 4 Cell and Molecular Biology Program, School of Life Sciences, Faculty of Science, The Chinese University of Hong Kong, Hong Kong SAR, China; tkyau@link.cuhk.edu.hk; 5 Department of Otorhinolaryngology, Head and Neck Surgery, Faculty of Medicine, The Chinese University of Hong Kong, Hong Kong SAR, China; janicelin@ent.cuhk.edu.hk; 6 Lee Kong Chian School of Medicine, Nanyang Technological University, Singapore 308232, Singapore; sunny.wong@ntu.edu.sg; 7 ProBioLife Limited, Hong Kong SAR, China

**Keywords:** gut microbiota, gut health, obesity, metabolic syndrome, insulin resistance, weight loss, diet, fruits and vegetables, probiotics, synbiotics

## Abstract

In view of the limited evidence showing anti-obesity effects of synbiotics via modulation of the gut microbiota in humans, a randomized clinical trial was performed. Assessment of the metabolic syndrome traits and profiling of the fecal gut microbiota using 16S rRNA gene sequencing in overweight and obese Hong Kong Chinese individuals before and after dietary intervention with an 8-week increased consumption of fruits and vegetables and/or synbiotic supplementation was conducted. The selected synbiotic contained two probiotics (*Lactobacillus acidophilus* NCFM and *Bifidobacterium lactis* HN019) and a prebiotic (polydextrose). Fifty-five overweight or obese individuals were randomized and divided into a synbiotic group (SG; n = 19), a dietary intervention group (DG; n = 18), and a group receiving combined interventions (DSG; n = 18). DSG showed the greatest weight loss effects and number of significant differences in clinical parameters compared to its baseline values—notably, decreases in fasting glucose, insulin, HOMA-IR, and triglycerides and an increase in HDL-cholesterol. DSG lowered *Megamonas* abundance, which was positively associated with BMI, body fat mass, and trunk fat mass. The results suggested that increasing dietary fiber consumption from fruits and vegetables combined with synbiotic supplementation is more effective than either approach alone in tackling obesity.

## 1. Introduction

The prevalence of obesity has increased substantially among Chinese adults during the past decades [1]. In Hong Kong, 29.9% of the population aged 15 to 84 was obese (BMI ≥ 25 kg/m^2^) [2], which is comparable to that in some Western countries using BMI ≥ 30 kg/m^2^ [3]. Previously, it was demonstrated that the prevalence of obesity in Hong Kong was greater than that in mainland China when BMI ≥ 25 kg/m^2^ was adopted [4]. The observed difference could be attributed to the early occurrence of westernization and urbanization in Hong Kong [5], which are important drivers of obesity [3]. Additionally, it could be due to the fact that Hong Kong citizens spend more time on sedentary activities than those from Western countries and mainland China [6].

Conventional non-surgical interventions using diet and exercise demonstrated limitations in achieving long-term weight loss [7]. Growing evidence has highlighted the importance of gut microbiota in the pathogenesis of obesity and its associated metabolic dysfunction [8]. Studies have shown that obese individuals harbor distinct gut microbiota compared with lean counterparts, and humanized mouse models revealed the possibility of gut microbiota as a causative factor in obesity [9,10]. Therefore, targeting the gut microbiota to reduce energy harvest from diet may be considered a promising approach to the treatment of obesity [11,12].

It is worth mentioning that a high-fiber diet, which is one of the popular diets used for weight loss, has been associated with an increase in microbiota-accessible carbohydrate-degrading bacteria and microbial diversity, which are essential to maintaining gut homeostasis [13,14,15,16,17]. Foods rich in dietary fiber (DF) such as fruits and vegetables (F&V) possess a wide range of phytochemicals that could also modulate the gut microbiota and bring anti-obesity, anti-inflammatory, and lipid-lowering effects [18]. Although high DF intake might contribute to weight loss via increased satiety [19], bloating, abdominal pain, and constipation could occur [20,21]. These gastrointestinal discomforts could hinder adherence to the feeding regimen in the long run, indicating that an effective yet comfortable dietary intervention is critical for wellbeing.

Dietary interventions in obesity using synbiotics, which are comprised of probiotic(s) and prebiotic(s), to re-establish gut homeostasis could facilitate weight loss [22]. Defined by the Food and Agriculture Organization of the United Nations and the WHO, probiotics are living microorganisms that when administered in sufficient amounts, confer beneficial health effects on the host by modulating the gut microbiota [23]. For example, patients with metabolic syndrome (MetS) experienced decreases in BMI, total cholesterol (TC), LDL-cholesterol, tumor necrosis factor α, and interleukin-6 after receiving fermented milk with *Bifidobacterium lactis* HN019 supplementation for 45 days compared with baseline and a control group [24]. Furthermore, it was found that when *Lactobacillus acidophilus* NCFM was administered to individuals with type 2 diabetes mellitus and impaired or normal glucose tolerance for four weeks, there was an increase in insulin sensitivity [25].

Prebiotic is defined as a substrate that is selectively utilized by host microorganisms, conferring a health benefit [26]. Polydextrose (PDX) is a highly branched and randomly bonded glucose polymer that has been suggested to possess prebiotic potential [27]. PDX might be useful to regulate blood glucose and lipid metabolism [27]. For example, reduced peak glucose and postprandial insulin responses resulted from the substitution of 30% of the available carbohydrates with PDX at breakfast and lunch in overweight men and women [28]. Moreover, an animal study showed that PDX enriched *Allobaculum* and *Bifidobacterium* in Western diet-fed mice in 14 days. A negative association between fasting triglycerides (TG) and TC and these two enriched genera was found, reflecting the hypolipidemic effect of PDX via regulation of the gut microbiota [29]. Taken together, it is worth investigating the inclusion of PDX in a synbiotic formulation containing *B. lactis* HN019 and *L. acidophilus* NCFM to target obesity in humans. Recently, a clinical trial has shown that a synbiotic supplement could increase the abundance of beneficial microbial species in obese subjects having low-carbohydrate, high-protein diets, yet no significant difference in weight loss was observed compared to a placebo group [30]. Nevertheless, human studies on the effects of combining synbiotics and other types of weight loss diets are limited and remain to be investigated.

The objective of this clinical trial was to evaluate the changes in gut microbiota and parameters of MetS in overweight and obese Hong Kong Chinese individuals before and after dietary intervention with an 8-week increased consumption of F&V and/or synbiotic supplementation. This clinical trial revealed that synbiotic supplementation would amplify the anti-obesity effects on body weight, BMI, and body fat mass as well as the improvement of MetS traits.

## 2. Materials and Methods

### 2.1. Study Participants

**Figure 1:**
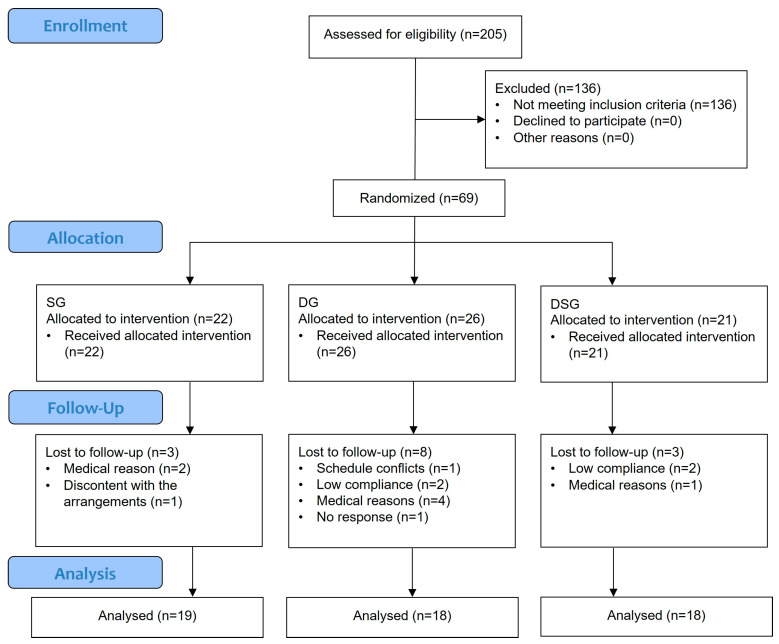
CONSORT flow diagram.

This study was an open-label, randomized, parallel-design clinical trial conducted in the Hong Kong Science Park. Overweight and obese Hong Kong Chinese individuals were recruited from January 2021 to October 2021 and randomly assigned to three different intervention groups. Sample size was determined using an online power calculator (www.clincalc.com/stats/samplesize.aspx) accessed on 11 December 2018. Based on the data of our pilot dietary intervention study, a minimum of 48 subjects were needed to reach 80% power with α = 0.05 to show an increase in alpha diversity from 3.74 (baseline) to 4.6 (endpoint) with standard deviation of 0.86. Both the researchers and participants recognized which intervention was being administered. All participants gave written informed consent. The Joint Chinese University of Hong Kong—New Territories East Cluster Clinical Research Ethics Committee approved the study protocol (Ref no.: 2019.165). The clinical trial has been registered at ClinicalTrials.gov (NCT no.: NCT05459909). The inclusion criteria were as follows: (1) aged between 20 and 65; (2) waist circumference ≥ 90 cm for men and ≥ 80 cm for women; and (3) BMI > 23 kg/m^2^. The major exclusion criteria were as follows: (1) practicing dietary restrictions; (2) using any drugs (e.g., cholesterol-lowering, anti-hypertensive, anti-inflammatory drugs, or Chinese medicine) within one week before the study commenced; (3) consuming laxatives, any gastrointestinal medication, probiotics, prebiotics, synbiotics, or antibiotics within one month before the study commenced; (4) smokers; (5) alcohol abusers; and (6) currently pregnant or lactating. Participation in the trial would be terminated immediately if any of the above exclusion criteria were met during the intervention. Participants were randomly assigned to three parallel groups for up to eight weeks: a synbiotic supplementation group (SG), a dietary intervention group (DG), or a dietary intervention with synbiotic supplementation group (DSG). An independent staff member performed simple randomization using computer-generated codes generated by www.randomization.com (accessed on 14 April 2021) to assign the participants to three different groups in a 1:1:1 ratio. The study flowchart and enrolment are shown in Figure 1. Apart from the baseline visceral fat rating, no significant difference was found in the baseline characteristics (Table 1).

### 2.2. Follow-Up Assessment and Synbiotic Supplement

Participants were followed up with by nutritionists every week until week 8. DG and DSG followed a one-goal weight loss program eating plan. The goal of the dietary intervention set for participants was to consume 30 ± 5 g DF from F&V per day based on the recommendation of the Healthy Eating Food Pyramid in Hong Kong (≥ 2 servings of fruits and ≥ 3 servings of vegetables). Participants were allowed to consume an ad libitum diet except for F&V intake. The dietary records provided to DG and DSG were checked every week by the nutritionists to ensure their compliance to the dietary intervention protocol. The synbiotic supplement provided to SG and DSG contained a mixture of 1 × 10^10^ CFU of probiotics (5 × 10^9^ CFU of *Bifidobacterium lactis* HN019, 5 × 10^9^ CFU of *Lactobacillus acidophilus* NCFM) and a prebiotic (1.7 g of polydextrose) packed in a sachet. The probiotics and prebiotic were purchased from DuPont Danisco, USA. SG and DSG were instructed to take two sachets in the morning and evening every day. Additionally, they were required to return all opened sachets to the nutritionists every week to ensure their adherence to the synbiotic supplementation protocol. Primary outcome measures were changes in body weight, BMI, and body fat mass. Secondary outcome measures were changes in MetS traits and gut microbiota profile. According to the International Diabetes Federation, an individual is qualified for the MetS if three out of five abnormal findings are present, including central obesity, decreased HDL-cholesterol (HDL-C), increased blood pressure, TG, and fasting glucose [31].

### 2.3. Body Composition and Metabolic Parameters

Body composition parameters were acquired before and after eight weeks of intervention by bioelectrical impedance analysis (BIA) using a TANITA MC-780 MA (TANITA, Tokyo, Japan). After the participant stands on the monitor, the TANITA BIA measures the whole-body composition by sending a safe, low-level electrical current from the feet to the legs and abdomen via the four metal electrodes in the scale’s footpads. The current passes quickly through the muscle tissue but meets more resistance when it encounters fat. This resistance is used for calculation of the body composition measurements by TANITA equations within 20 s. The measurements include body weight, body fat mass, muscle mass, bone mass, and body water. The accuracy of the body composition report is at least 97% (TANITA, 2022).

Blood samples after an overnight fast were collected and examined at baseline and at the end of study by KingMed Diagnostic (Hong Kong) Limited. Plasma glucose levels (enzymatic reference method with hexokinase) were measured on a Roche Cobas C-701. Serum TC (enzymatic method), HDL-C (polyanion precipitation), TG (enzymatic colorimetric test), and C-reactive protein (particle-enhanced immunoturbidimetric assay) were measured on a Roche Cobas C-702. Serum insulin (human insulin immunoassay) was measured on a Roche Cobas E411. The reference values of clinical markers for healthy individuals are shown in Table A1, and the reference values of fasting glucose for the classification of non-diabetes mellitus (DM), impaired fasting glucose, and DM are displayed in Table A2. Homeostasis model assessment of insulin resistance (HOMA-IR) was calculated as (fasting insulin [μlU/mL] × fasting glucose [mmol/L])/22.5 [32]. To depict the improvement in metabolic health among participants after intervention, evaluation of the number of risk factors for MetS was performed in terms of abnormal fasting glucose, HDL-C, and TG levels. Normal levels of these three parameters represent zero risk factor (0MS), whereas 1MS, 2MS, and 3MS indicate that the participants possessed one, two, and three MetS risk factors, respectively.

### 2.4. Microbial DNA Extraction and the 16S rRNA Gene Sequencing

Fecal samples from the participants were collected using a fecal collection kit and delivered on ice bags before and after intervention. Samples were frozen and stored at −20 °C until use. DNA was extracted using a QIAamp Fast DNA Stool Mini Kit (Qiagen, Valencia, CA, USA) according to the manufacturer’s instructions. The purity of the DNA samples was quantified by measuring the A260/280 ratio with the Nanodrop OneC spectrophotometer (Thermo Fisher Scientific, Waltham, MA, USA). DNA samples were frozen and stored at −20 °C until use. 110 DNA samples were sent to Novogene Bioinformatics Technology Co., Ltd. (Beijing, China) for paired-end 16S rRNA gene sequencing. The quality and quantity of samples were checked using an Agilent 5400 Bioanalyzer (Agilent, Santa Clara, CA, USA) before library preparation. The V4 region of the 16S rRNA gene was amplified with PCR using 515F (5′-GTGCCAGCMGCCGCGGTAA-3′) and 806R (5′-GGACTACHVGGGTWTCTAAT-3′) primers connected with barcodes [33]. 16S rRNA gene sequencing (2 × 250 bp PE V4) was performed on an Illumina Novaseq 6000 platform.

### 2.5. Taxonomic Classification and Bioinformatics Analysis

The raw reads were processed and analyzed with QIIME 2 2022.8 for taxonomy classification and bioinformatic analysis [34]. The raw reads were primer-trimmed by cutadapt and joined with DADA2 [35]. Taxonomy was assigned to amplicon sequence variants (ASVs) on the Greengenes v.13_8 99% OTUs reference by the sklearn classifier. ASV with less than 10 reads or present in only one sample was removed by the filter features. Furthermore, unclassified reads at the phylum or genus level were discarded. The sequencing reads of the samples were normalized by rarefaction. Relative abundance was used for statistical analysis. The alpha-diversity and beta-diversity were measured using the core-metrics-phylogenetic pipeline.

### 2.6. Data Analysis

Statistical significances were assessed by paired two-tailed *t*-test or one-way ANOVA followed by Tukey’s post hoc test using GraphPad Prism 9 (GraphPad Software, La Jolla, CA, USA). Differences are considered significant when the *p*-values are below 0.05. Alpha-diversity was assessed using the Shannon diversity index and tested using a paired two-tailed *t*-test. Beta-diversity was measured by PERMANOVA analysis with 9999 permutations and was visualized using a principal coordinate analysis (PCoA) plot based on weighted UniFrac distances. Linear discriminant analysis effect size (LEfSe) analysis was performed to identify significantly altered bacteria before and after intervention [36], with the threshold on the logarithmic score of linear discriminant analysis set at 2.0. Pearson correlation analysis and the construction of heatmaps were performed in RStudio version 2022.07.0 using the corrplot and Hmisc packages.

## 3. Results

### 3.1. Body Composition

**Figure 2:**
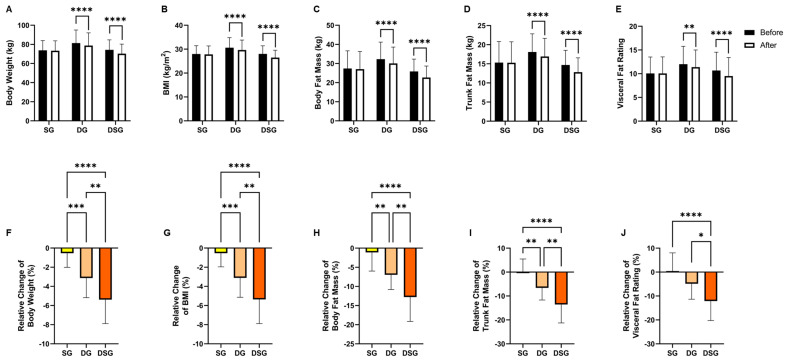
Changes in anthropometric measures. (**A**) Body weight, (**B**) BMI, (**C**) body fat mass, (**D**) trunk fat mass, and (**E**) visceral fat rating before and after intervention. Relative change in (**F**) body weight, (**G**) BMI, (**H**) body fat mass, (**I**) trunk fat mass, and (**J**) visceral fat rating after intervention compared to baseline. Data are expressed as mean ± SD. Paired two-tailed *t*-test and one-way ANOVA followed by Tukey’s post hoc test were used for intragroup comparison and intergroup comparison, respectively. * *p* < 0.05, **: *p* < 0.01, ***: *p* < 0.001, ****: *p* < 0.0001.

Intragroup comparisons in anthropometric measures revealed that SG did not exhibit any significant changes. On the contrary, both DG and DSG showed significant reductions in body weight, BMI, body fat mass, trunk fat mass, and visceral fat rating compared to baseline (Figure 2A–E). Regarding intergroup comparisons, the relative decrease in body weight, BMI, body fat mass, and trunk fat mass were significantly greater for both DG and DSG compared to SG. The relative decrease in these four obesity indices was significantly more pronounced in DSG compared to that in DG (Figure 2F–I). The relative decrease in visceral fat rating was significantly greater in DSG compared to that in SG and DG (Figure 2J).

**Figure 3:**
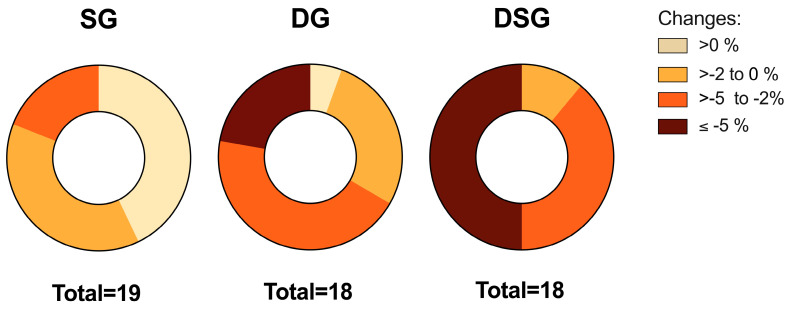
Distribution of the relative change in body weight among groups.

It has been proposed that 5% weight loss could be defined as a clinically significant weight loss if maintained after one year of treatment [37]. Variation in the distribution of weight loss was observed among groups. Comparatively, no participants lost more than 5% of their initial weight in SG, whereas approximately 22% and 50% of the participants in DG and DSG achieved weight loss of at least 5%, respectively (Figure 3).

### 3.2. Glycemic Parameters

**Figure 4:**
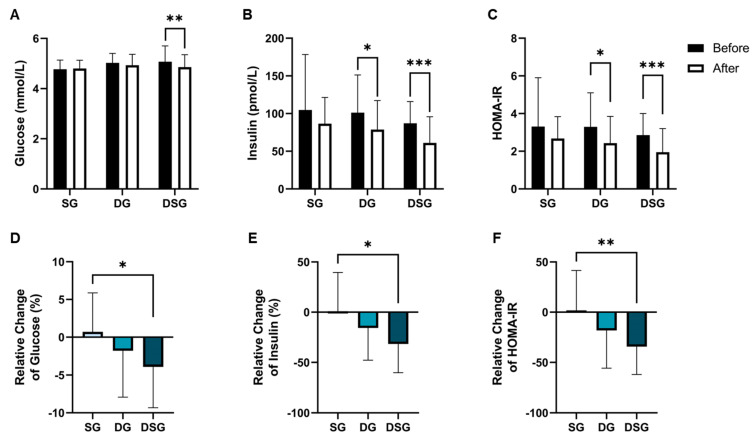
Changes in glycemic parameters. (**A**) Fasting glucose, (**B**) fasting insulin, and (**C**) HOMA-IR before and after intervention. Relative change in (**D**) fasting glucose, (**E**) fasting insulin, and (**F**) HOMA-IR after intervention compared to baseline. Data are expressed as mean ± SD. Paired two-tailed *t*-test and one-way ANOVA followed by Tukey’s post hoc test were used for intragroup comparison and intergroup comparison, respectively. *: *p* < 0.05, **: *p* < 0.01, ***: *p* < 0.001. HOMA-IR: Homeostasis model assessment of insulin resistance.

**Figure 5:**
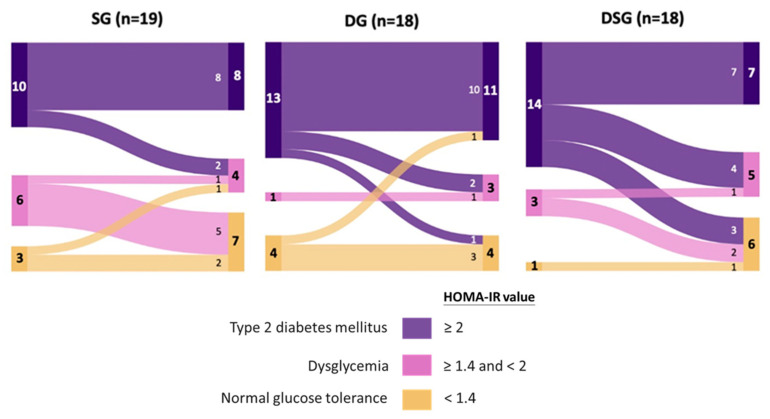
Sankey diagrams showing the flow of homeostasis model assessment of insulin resistance (HOMA-IR) among participants after intervention.

Compared to baseline, a significant decrease in fasting glucose was shown in DSG but not in SG and DG (Figure 4A). The fasting insulin level and HOMA-IR were significantly lowered in DG and DSG compared to baseline (Figure 4B,C). Although statistical significance was not shown, a decreasing trend in fasting insulin was shown in SG (Figure 5B). There was a significantly greater relative decrease in fasting glucose, fasting insulin, and HOMA-IR in DSG compared to SG (Figure 4D–F).

Optimal cut-offs of HOMA-IR of 1.4 and 2.0 were proposed to discriminate dysglycemia from normal glucose tolerance and T2DM from non-DM, respectively, in Hong Kong Chinese participants [38]. Given the fact that our recruited participants were non-DM, these optimal HOMA-IR cut offs were used to predict their risk of dysglycemia and T2DM before and after intervention. It could be seen that the majority of participants were prone to developing T2DM at baseline, with a limited number of participants belonging to the category of normal glucose tolerance (Figure 5). Interestingly, half of the participants with HOMA-IR greater than or equal to 1.4 in DSG experienced improved glycemic status, which was not observed in SG and DG.

### 3.3. Plasma Lipids and C-Reactive Protein

**Figure 6:**
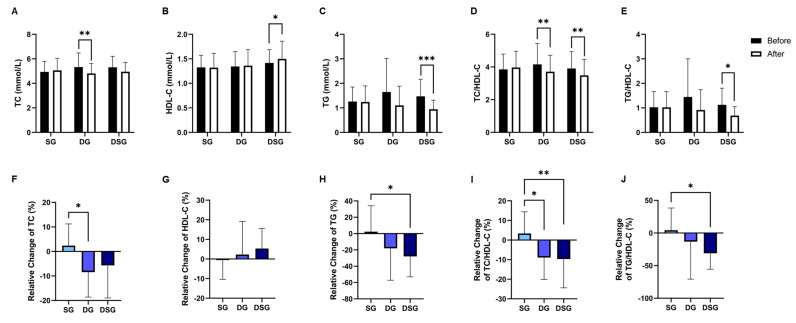
Changes in lipid parameters. (**A**) TC, (**B**) HDL-C, (**C**) TG, (**D**) TC/HDL-C, and (**E**) TG/HDL-C before and after intervention. Relative change in (**F**) TC, (**G**) HDL-C, (**H**) TG, (**I**) TC/HDL-C, and (**J**) TG/HDL-C after intervention compared to baseline. Data are expressed as mean ± SD. Paired two-tailed *t*-test and one-way ANOVA followed by Tukey’s post hoc test were used for intragroup comparison and intergroup comparison, respectively. *: *p* < 0.05, **: *p* < 0.01, ***: *p* < 0.001. TC: total cholesterol; HDL-C: HDL-cholesterol; TG: triglycerides.

**Figure 7:**
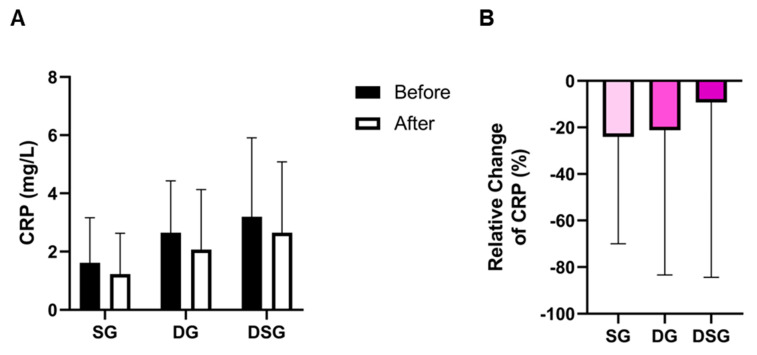
(**A**) Intragroup and (**B**) intergroup comparison of C-reactive protein (CRP). Data are expressed as mean ± SD. Paired two-tailed *t*-test and one-way ANOVA followed by Tukey’s post hoc test were used for intragroup and intergroup comparison, respectively.

TC was significantly lowered in DG compared to baseline (Figure 6A). DSG had a significantly higher HDL-C and TG compared to baseline (Figure 6B,C). The TC/HDL-C and TG/HDL-C decreased significantly in DSG compared to baseline (Figure 6D,E). Compared to SG, a significantly greater relative decrease in TC was found in DG (Figure 6F). However, there was no significant difference in the relative change in HDL-C between the three groups (Figure 6G). The relative decrease in TG was significantly greater in DSG compared to SG (Figure 6H). Compared to SG, a significant relative decrease in TC/HDL-C and TG/HDL-C was demonstrated in DSG (Figure 6I,J). No significant difference in C-reactive protein (CRP) was found in the intragroup or intergroup comparison (Figure 7).

### 3.4. Metabolic Syndrome Parameters

**Figure 8:**
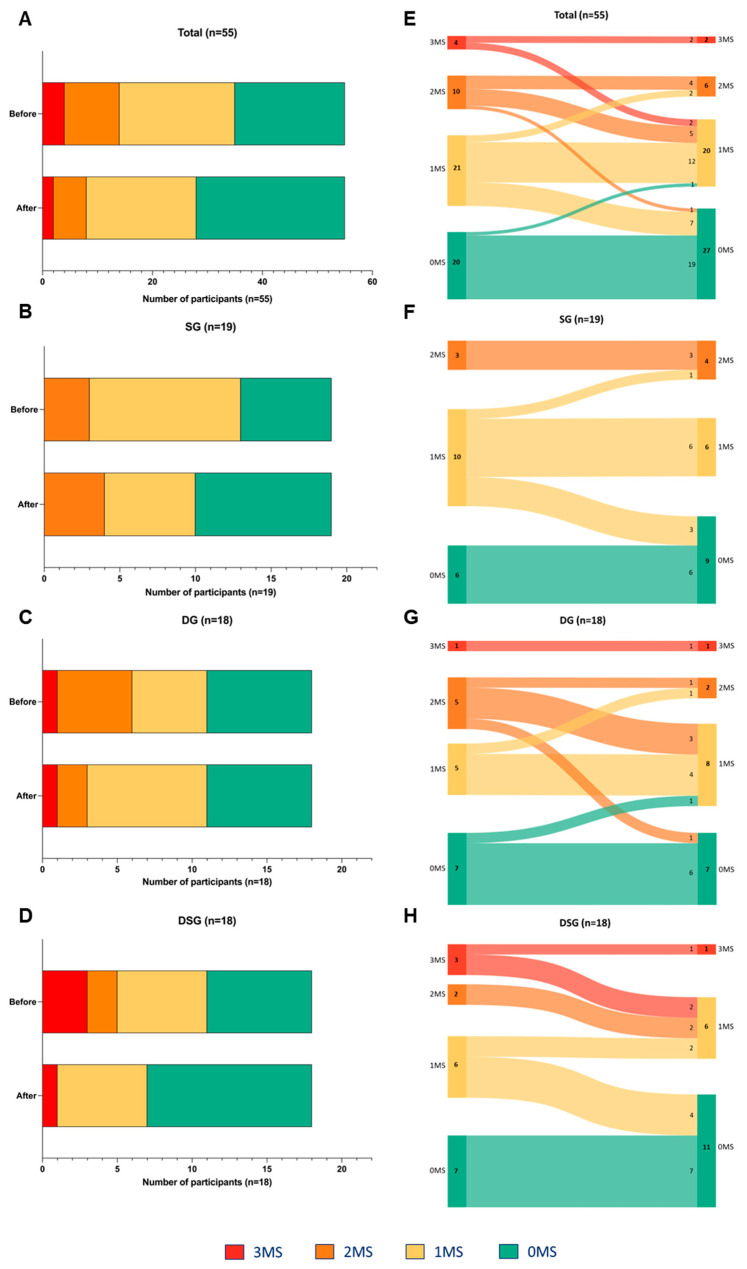
Changes in the number of MetS traits among (**A**) all, (**B**) SG, (**C**) DG, and (**D**) DSG participants after intervention. Sankey diagrams showing the flow of MetS traits among (**E**) all, (**F**) SG, (**G**) DG, and (**H**) DSG participants after intervention. MS represents the MetS traits including abnormal fasting glucose, HDL-C, and TG levels.

The risk factors for MetS among participants were assessed based on abnormal fasting glucose, HDL-C, and TG levels. Normal levels of these three parameters represent zero risk factor (0MS). There was a decreasing trend in the number of participants with three risk factors (3MS) and two risk factors (2MS). Moreover, an increasing trend in the number of participants with 0MS was observed after intervention (Figure 8A). Differential alteration in the MetS risk factors was demonstrated in different interventions. In SG, the population having one risk factor (1MS) decreased, but that of 0MS and 2MS increased (Figure 8B). In DG, the number of participants with 3MS and 0MS remained unchanged. However, there was a decreased and increased number of participants with 2MS and 1MS, respectively (Figure 8C). In DSG, the population of 3MS and 2MS decreased, and that of 1MS remained unchanged. Moreover, there were more participants with 0MS (Figure 8D).

Sankey diagrams were used to visualize the flow of participants from one category to the others. The participants tended to experience improved metabolic health after intervention (Figure 8E). Improvement in MetS parameters among participants after intervention was mostly seen in DSG (n = 8), followed by DG (n = 4) and SG (n = 3) (Figure 8F–H).

### 3.5. Sequencing Quality and Taxonomic Composition of Gut Microbiota

**Figure 9:**
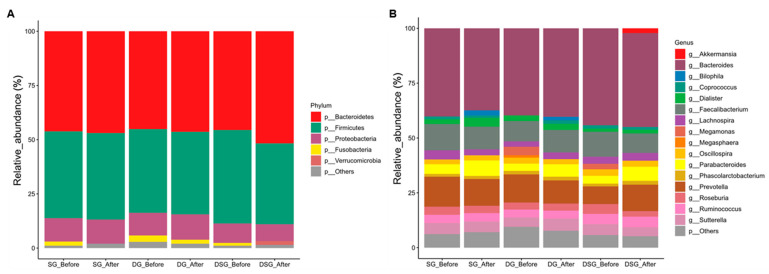
Taxonomic composition of gut microbiota before and after intervention. Mean relative abundance (%) of (**A**) phyla and (**B**) genera before and after intervention. Relative abundance of phyla and genera with less than 1% are regarded as others.

After filtering the low-quality reads, chimera removal, and denoising, a total of 18,722,472 reads was acquired from 110 samples, with an average of 170,204 reads per sample. More than 97% of the sequencing reads from each sample displayed a Phred quality score of 20. The composition and diversity of gut microbiota of participants before and after intervention were assessed. Bacteria with top 5 and 16 relative abundances at the phylum (Figure 9A) and genus (Figure 9B) levels, respectively, are shown. Firmicutes and Bacteroidetes account for the most abundant bacterial phyla in the gut (Figure 9A). We observed heterogeneity in the gut microbiota profile at the genus level in participants before and after intervention (Figure 9B).

### 3.6. Firmicutes-to-Bacteroidetes Ratio, Alpha-Diversity, and Beta-Diversity

**Figure 10:**
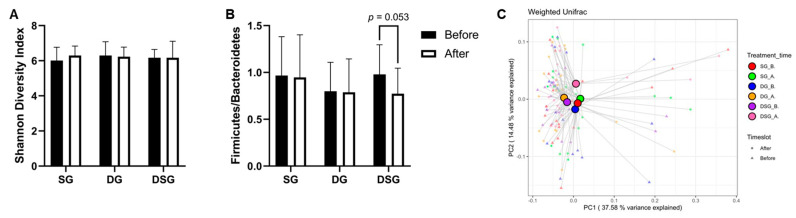
Firmicutes-to-Bacteroidetes ratio (F/B), alpha-diversity, and beta-diversity before and after intervention. (**A**) F/B, (**B**) Shannon diversity index, and (**C**) principal coordinate analysis (PCoA) of gut microbiota structures.

Although there was no significant difference in Firmicutes-to-Bacteroidetes ratio (F/B) and Shannon diversity index between the three groups (Figure 10A,B), it is noteworthy that F/B tended to decrease in DSG after intervention (*p* = 0.053). The beta diversity was evaluated by the weighted-UniFrac PCoA. The centroids of gut microbiota composition before and after intervention in SG and DG clustered together on the PCoA plot but were distinct from that of DSG after intervention (Figure 10C).

### 3.7. Linear Discriminant Analysis Effect Size Analysis of Gut Microbiota

**Figure 11:**
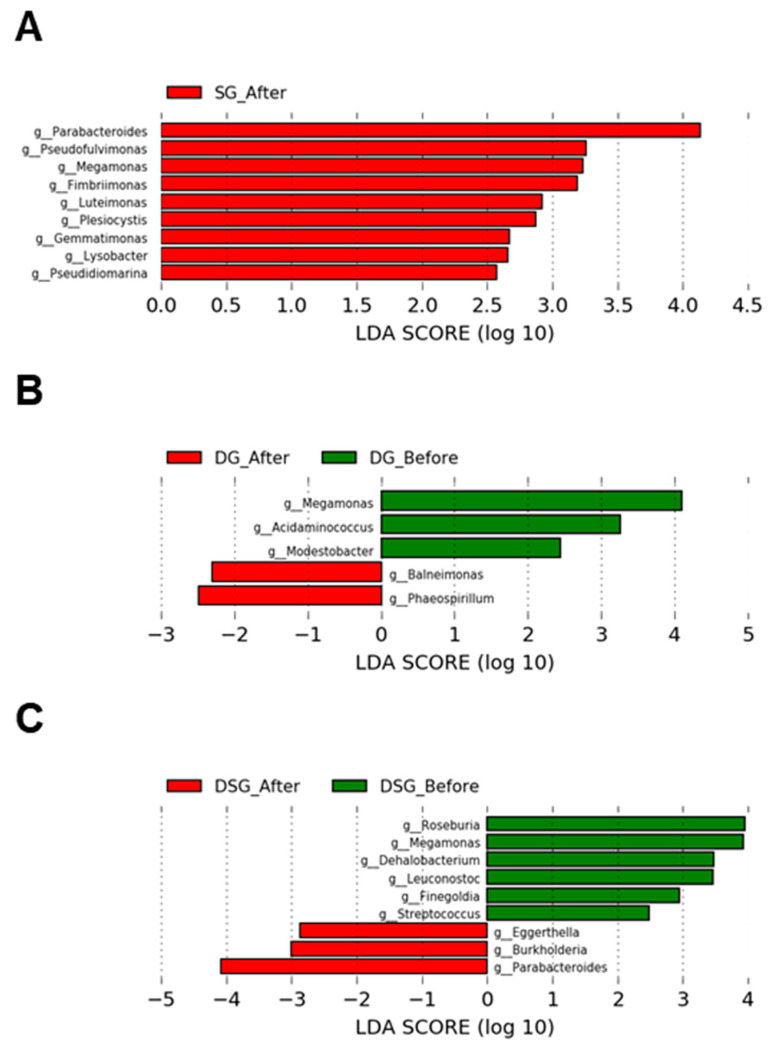
LEfSe plots of genera before and after intervention in the (**A**) SG, (**B**) DG, and (**C**) DSG.

In SG, the relative abundance of the genera *Parabacteroides*, *Pseudofluvimonas*, *Megamonas*, *Fimbriimonas, Luteimonas*, *Plesiocytis*, *Gemmatimonas*, *Lysobacter*, and *Pseudidiomarina* was significantly higher after intervention (Figure 11A). In DG, the relative abundance of the genera *Baleimonas* and *Phaeospirillum* was significantly increased after intervention. Compared to baseline, DG had significantly lower levels of the genera *Megamonas*, *Acidaminococcus*, and *Modestobacter* (Figure 11B). In DSG, the relative abundance of the genera *Eggerthella*, *Burkholderia*, and *Parabacteroides* was significantly higher after intervention. Compared to baseline, DSG had significantly lower levels of the genera *Megamonas*, *Roseburia*, *Leuconostoc*, *Dehalobacterium*, *Finegoldia*, and *Streptococcus* (Figure 11C).

### 3.8. Correlation Analysis among Body Composition Parameters, Metabolic Biomarkers, and Gut Microbiota

**Figure 12:**
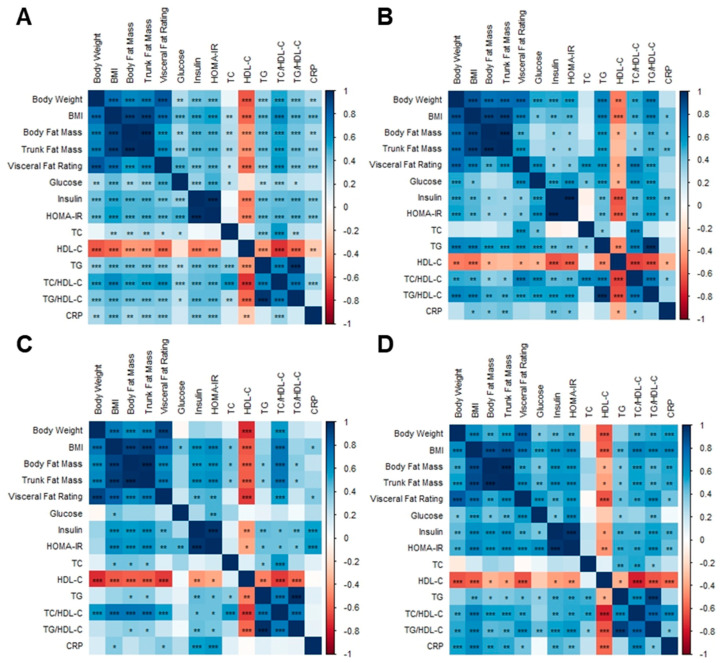
Associations between body composition parameters and metabolic biomarkers. (**A**) Overweight and obese Hong Kong Chinese individuals, (**B**) SG, (**C**) DG, and (**D**) DSG. Blue squares indicate positive correlations, and red squares indicate negative correlations. *: *p* < 0.05, **: *p* < 0.01, ***: *p* < 0.001.

The BMI, body fat mass, trunk fat mass, and visceral fat rating of overweight and obese Hong Kong Chinese individuals were positively correlated with glucose, insulin, HOMA-IR, TC, TG, TC/HDL-C, TG/HDL-C, and CRP, whereas they were negatively correlated with HDL-C (Figure 12A). Fasting glucose was positively correlated with body weight, visceral fat rating, and insulin in SG and DSG (Figure 12B,D). However, these correlations were not shown in DG (Figure 12C).

**Figure 13:**
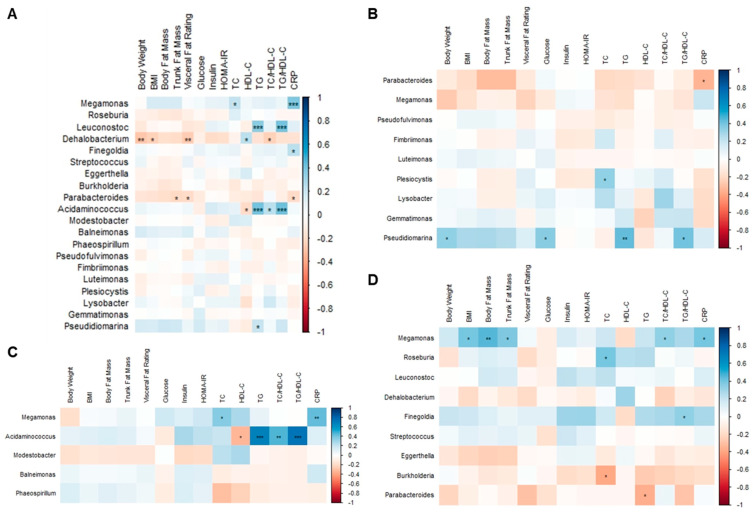
Associations between body composition parameters, metabolic biomarkers, and gut microbiota at the genus level. (**A**) Overweight and obese Hong Kong Chinese individuals, (**B**) SG, (**C**) DG, and (**D**) DSG. Blue squares indicate positive correlations, and red squares indicate negative correlations. *: *p* < 0.05, **: *p* < 0.01, ***: *p* < 0.001.

In overweight and obese Hong Kong Chinese individuals, *Megamonas* abundance was positively correlated with TC and CRP (Figure 13A). *Leuconostoc* and *Acidaminococcus* abundance were positively correlated with TG and TG/HDL-C. Additionally, there was a positive correlation between *Finegoldia* abundance and CRP. Negative correlations were found between *Dehalobacterium* abundance and body weight, BMI, visceral fat rating, and TC/HDL-C, as well as between *Parabacteriodes* abundance and trunk fat mass, visceral fat rating, and CRP.

In SG, it was shown that *Parabacteroides* abundance was negatively correlated with CRP. Positive correlations were demonstrated between *Plesiocytis* abundance and TC and between *Pseudidiomarina* abundance and body weight, fasting glucose, TG, and TG/HDL-C (Figure 13B). In DG, *Megamonas* abundance was positively correlated with TC and CRP. Moreover, *Acidaminococcus* abundance was positively correlated with TG, TC/HDL-C, and TG/HDL-C but negatively correlated with HDL-C (Figure 13C). In DSG, positive correlations were found between *Megamonas* abundance and BMI, body fat mass, trunk fat mass, TC/HDL-C, and CRP and between *Roseburia* abundance and TC. Furthermore, *Finegoldia* abundance was positively correlated with TG/HDL-C. Additionally, negative correlations were found between *Burkholderia* abundance and TC and between *Parabacteroides* abundance and TG (Figure 13D).

## 4. Discussion

The present study was an open-label randomized clinical trial aimed to evaluate the effects of a synbiotic supplement containing *B. lactis* HN019, *L. acidophilus* NCFM, and PDX on the anthropometric indices, obesity-related biochemical markers, and gut microbiota in overweight and obese Hong Kong Chinese participants in a weight loss program. Although the individual prebiotic or probiotic component of synbiotic has been demonstrated to improve markers of MetS [24,25,29], the application of this synbiotic supplement in obesity management has not been observed. F&V, an important source of DF, are consumed at an inadequate level by the Hong Kong population. Our weight loss program was an eating plan emphasizing increased F&V consumption based on the Healthy Eating Food Pyramid in Hong Kong [39]. A lack of DF would increase the risk of gut barrier dysfunction due to intestinal mucus erosion, which has a positive relationship with obesity and MetS [40,41].

An increased DF diet can lead to the occurrence of bloating, abdominal pain, and constipation [20,21], so we considered administering a synbiotic to prevent the potential gastrointestinal discomfort of our weight loss diet. It has been reported that our selected synbiotic formulation could be an option for chronic constipation treatment, because it could significantly the shorten colonic transit time [42]. It was observed that gastrointestinal discomfort, such as constipation, diarrhea, bloating, and gas, was reported by some participants in DG when adapting to the increased DF intake at the early phase of the intervention. This phenomenon was not observed among participants in DSG. They could increase their consumption of DF at ease without feeling any discomfort.

The results of the current trial revealed that the use of a synbiotic supplement alone had no significant effects on body composition and metabolic biomarkers in overweight and obese individuals. In line with our results, a meta-analysis reported that oral supplementation with synbiotics has a mild effect on the reduction in waist circumference yet no effect on body weight or BMI [43]. In contrast, a recent study found a significant decrease in body weight, BMI, and body fat percentage after 12 weeks of synbiotic supplementation (a mixture of *L. paracasei*, *B. longum*, *B. breve*, inulin, and fructooligosaccharide) in Thai obese adults [44]. Therefore, it seems that a prolonged intervention period might be needed to demonstrate the weight loss effects of synbiotic on obesity.

One of the interesting points of our study was the optimal weight loss performance in terms of body weight, BMI, body fat mass, trunk fat mass, and visceral fat rating in DSG compared to SG and DG. Our findings coincide with a previous report showing that synbiotic supplementation plus weight loss diet had more synergistic effects on body weight and BMI compared to the placebo group [45]. Only DSG lost 5% body weight on average at the end of the intervention, which has the prospect of fulfilling the criterion of clinically significant weight loss if maintained after one year of treatment [37]. Even though it is generally recognized that greater weight loss would attain better health performance, this 5% weight loss acts as a benchmark for successful individuals’ response to the treatment [37]. Modest weight loss of between 5% and 10% was found to be associated with improvement in CVD risk factors, including decrease in blood pressure, TG, and increase in HDL-C [46,47]. Additionally, it was demonstrated that improvement of HbA1c, fasting glucose, and insulin sensitivity began at ≥ 2% weight loss [47,48].

DF has been identified as the only nutritional variable to predict health outcomes, and its adequacy should be highly emphasized in a diet-based weight loss program [49]. An analysis of biochemical variables showed that TC and TC/HDL in DG were significantly lower than that in SG, implying that our weight loss diet would be beneficial to cardiovascular health. Additionally, our study showed that when a synbiotic supplement is taken with a DF-enriched diet, the decreases in fasting glucose, fasting insulin, and HOMA-IR would be significantly greater than when synbiotic supplementation is used alone. It is possible that increased consumption of DF from F&V is a key source of non-digestible ingredients to modify the gut microbiota to improve body composition and metabolic health [49]. Furthermore, DF could be fermented by the gut microbiota and probiotic bacteria to produce short-chain fatty acids (SCFAs), leading to an improvement in glucose homeostasis and insulin sensitivity [50,51].

Although reduced fasting glucose, fasting insulin, HOMA-IR, and TG were observed compared to baseline in DSG, no significant difference in these metabolic outcomes was detected between DG and DSG. This is understandable, because the level of these biochemical indices fell into a normal range after intervention in DG and DSG. Our findings are similar to another study in which these metabolic outcomes were not significantly altered when a very-low-calorie ketogenic diet was accompanied with synbiotic supplementation [52]. On top of the weight loss diet, it is speculated that the profound anti-obesity effects shown in DSG are indirectly mediated by PDX. PDX is a source of added fiber that the US Food and Drug Administration has approved for DF status. Although PDX provides 1 kcal/g, it is able to lower food intake, decrease body weight, and increase satiety [53]. Previously, PDX was shown to enhance the intestinal barrier function in patients with severe acute pancreatitis, probably due to the stimulation of mucin production, which strengthens intestinal epithelial tight junctions [54]. Since BMI, body fat mass, and visceral adiposity have been positively associated with increased intestinal permeability [55,56], a substantial improvement in intestinal permeability resulting from PDX-containing synbiotic supplementation might be a possible mechanism to explain the greatest reduction in obesity observed in DSG.

The pronounced weight loss effects demonstrated in DSG compared to DG could be modulated by the gut microbiota because of the synergistic action of the synbiotic supplement and DF. It has been suggested that there is a positive association between obesity and the F/B [57], but some studies found no association [58]. Our results found no significant difference in F/B in overweight and obese individuals after weight loss in SG and DG. Interestingly, the decrease in F/B in DSG was near to significant (*p* = 0.053). This suggests that increasing F&V consumption with synbiotic supplementation can highly modulate the gut microbiota, and it acts as a promising gut microbiota-targeted weight loss strategy.

LEfSe analysis showed that the genus *Megamonas* increased in SG but decreased in DG and DSG after the intervention, although the three intervention groups displayed decreasing trends in body weight, BMI, and body fat mass. Currently, there is no clear consensus on the role of *Megamonas* in obesity, and its abundance in obese individuals remains contradictory. It was reported that a lower *Megamonas* abundance was found in obese Mexican adolescents compared to normal weight adolescents [59]. However, previous clinical trials found that the level of *Megamonas* was significantly higher in obese Taiwanese adults [60], Mexican children [61], and Chinese children [62] compared to their non-obese counterparts. Recently, *Megamonas* was demonstrated to be positively associated with body fat mass in Italian adults [63].

It is worth highlighting that different associations of *Megamonas* and body composition parameters and metabolic biomarkers were established. An important finding is that *Megamonas* is significantly and positively associated with BMI, body fat mass, and trunk fat mass in DSG but not in DG, suggesting that our synbiotic supplement facilitates *Megamonas* to become a key genus responsible for amplifying the anti-obesity effects of our weight loss diet. On top of the significant decrease in the genus *Megamonas*, it is speculated that the significant increase in the genus *Parabacteroides* might contribute to the greatest weight loss effects observed in DSG after intervention. We speculate that the anti-obesity response mediated by *Parabacteroides* might be diet-specific, which is a diet with increased DF from F&V. Although *Parabacteroides* was also enriched after SG, no significant weight loss effect resulted, because the DF intake of SG participants was not adjusted. *Parabacteroides* has been identified as an acetate-producing bacteria [64]. Acetate is the most abundantly found SCFA. It could promote the secretion of gut peptides, including glucagon-like peptide-1 and peptide YY, which are involved in the reduction in appetite and improvement in dysglycemia and insulin resistance [65,66]. Although the fecal SCFAs of the participants were not measured, it is possible that there was an increase in acetate in parallel with the elevated relative abundance of *Parabacteroides* resulting from increased F&V with synbiotic supplementation.

We were unable to detect the two probiotic species in the fecal samples of SG and DSG participants due to the limitation of 16S rRNA gene sequencing, which can only detect the identity of bacteria up to the genus level. Although the sample size was small and the study period was relatively short, the difference in weight loss percentage between DG and DSG was noteworthy and clinically meaningful. Research has suggested that any type of weight loss diet can be effective if participants demonstrate strong adherence with persistence and enthusiasm [67]. The prospect of achieving clinically meaningful weight loss among most of the participants in DSG could be attributed to the simplicity of our intervention (increasing DF from F&V with synbiotic supplementation), leading them to demonstrate their strong adherence to the program. It is of utmost importance to establish a manageable weight loss diet for overweight and obese individuals in light of their personal and cultural backgrounds for long-term adherence and weight maintenance.

The absence of a placebo group is one of the limitations of this study, since we are unable to show whether the same analyzed parameters would undergo modifications if participants had not undergone an intervention. The age and gender effects on the overall results could not be determined because of the relatively small sample size and uneven gender distribution of participants. The short study duration might explain the insignificant weight loss and metabolic health improvement after synbiotic supplementation compared to baseline. Since there could be bias in the self-reported dietary data, it is hoped that standardized catering services could be offered to participants to ensure similar dietary intake between DG and DSG. We suggest assessing the serum zonulin level and fecal SCFAs to understand the effects of our weight loss program on intestinal permeability and microbial fermentation, respectively. As DSG showed a significant decrease in fasting glucose, fasting insulin, and HOMA-IR compared to its baseline values, it would be meaningful to investigate the hypoglycemic effect of our weight loss program on type 2 DM patients who are overweight or obese.

## 5. Conclusions

This study presents the first investigation into the effects of increasing F&V consumption with synbiotic supplementation on overweight and obese Hong Kong Chinese individuals. This information is useful in demonstrating a simple and sustainable strategy to increase DF intake to the daily recommended level while being accompanied by a weight loss effect. Increased F&V should be targeted as the most important nutritional variable for primary consideration in a dietary intervention for weight loss. Relative to synbiotic supplementation and dietary intervention alone, the combination of these two components seems to be more effective in alleviating obesity and its associated MetS traits. Furthermore, DSG decreased the abundance of *Megamonas*, which was positively associated with BMI, body fat mass, and trunk fat mass. Our data support the fact that the synergistic action of our weight loss diet and synbiotic supplement could be linked to the favorable change in gut microbiota genera associated with obesity indicators.

## Figures and Tables

**Table 1:** Baseline characteristics of study participants.

Parameters	SG	DG	DSG	*p*
n	19	18	18	
Men	4	4	6	
Women	15	14	12	
Age (years)	40.6 ± 10.0	42.0 ± 8.85	44.6 ± 13.4	0.528
Body Weight (kg)	73.8 ± 10.2	81.3 ± 13.7	74.3 ± 10.5	0.095
BMI (kg/m^2^)	28.0 ± 3.5	30.6 ± 4.2	28.0 ± 3.4	0.059
Body Fat Mass (kg)	27.4 ± 9.3	32.3 ± 8.9	25.9 ± 6.5	0.062
Trunk Fat Mass (kg)	15.3 ± 5.5	18.1 ± 4.8	14.7 ± 3.8	0.085
Visceral Fat Rating	10.1 ± 3.5	12.0 ± 3.8	10.7 ± 3.9	0.028
Glucose (mmol/L)	4.8 ± 0.4	5.0 ± 0.4	5.1 ± 0.6	0.124
Insulin (pmol/L)	104.8 ± 73.6	101.2 ± 50.0	87.1 ± 28.8	0.584
HOMA-IR	3.3 ± 2.6	3.3 ± 1.8	2.9 ± 1.1	0.726
TC (mmol/L)	4.9 ± 0.9	5.3 ± 1.2	5.3 ± 0.9	0.398
HDL-C (mmol/L)	1.3 ± 0.2	1.3 ± 0.3	1.4 ± 0.3	0.575
TG (mmol/L)	1.3 ± 0.6	1.7 ± 1.4	1.5 ± 0.7	0.453
TC/HDL-C	3.9 ± 0.9	4.2 ± 1.3	3.9 ± 1.0	0.671
TG/HDL-C	1.0 ± 0.6	1.5 ± 1.6	1.1 ± 0.7	0.453
CRP (mg/L)	1.7 ± 1.5	2.7 ± 1.7	3.2 ± 2.7	0.071

One-way ANOVA followed by Tukey’s post hoc test was used for intergroup comparison. Data are expressed as mean ± SD. HOMA-IR: homeostasis model assessment of insulin resistance; TC: total cholesterol; HDL-C: HDL-cholesterol; TG: triglycerides; CRP: C-reactive protein.

## Data Availability

The microbiome sequencing data have been deposited to the NCBI Sequence Read Archive (SRA) under BioProject accession no. PRJNA1015382. Anonymized data presented in the manuscript and protocol will be made available upon reasonable request to the corresponding author.

## Appendix A

**Table A1:** Reference values of clinical markers for healthy individuals provided by KingMed Diagnostics (Hong Kong) Limited.

Clinical Marker	Reference Value
Total cholesterol	<5.2 mmol/L
HDL-cholesterol	>1.3 mmol/L
Triglycerides	<1.7 mmol/L
C-reactive protein	<5 mg/L
Fasting insulin	18–173 pmol/L
Fasting glucose	<5.6 mmol/L

**Table A2:** Reference values of fasting glucose for classification of non-diabetes mellitus (DM), impaired fasting glucose (IFG), and DM provided by KingMed Diagnostics (Hong Kong) Limited, with reference to the American Diabetes Association (ADA) criteria.

Clinical Marker	Non-DM	IFG (Prediabetes)	DM
Fasting glucose	<5.6 mmol/L	5.6–6.9 mmol/L	>6.9 mmol/L
